# S-decorated Mo_2_C as efficient catalyst for Li–O_2_ battery system

**DOI:** 10.1039/d5ra02021b

**Published:** 2025-07-08

**Authors:** Yanhong Ding, Zhichao Gao, Rongpeng Lin, Yong Cao, Haoyang Liu, Yulin Zhou, Haifeng Xu, Jiayi Liu, Fangqi Ren, Yirong Zhu

**Affiliations:** a College of Materials and Advanced Manufacturing, Hunan University of Technology Zhuzhou 412007 People's Republic of China zhuyirong2004@163.com

## Abstract

Lithium–oxygen (Li–O_2_) batteries are considered an important candidate for the next generation of energy storage systems due to their ultra-high theoretical energy density (11 586 mA h g^−1^), but their slow kinetic reactions, high overpotential and cyclic instability seriously limit their practical applications. In this study, sulfur modified Mo_2_C (S@Mo_2_C) cathode materials were prepared by hydrothermal synthesis by sulfur (S) doping to optimize the electronic structure and catalytic activity of Mo_2_C (Mo_2_C). Experiments show that S@Mo_2_C exhibits significantly improved electrochemical performance compared to commercial Mo_2_C: its specific capacity is up to 3955 mA h g^−1^ (commercial material only 508 mA h g^−1^), the charge and discharge overpotential is reduced to 0.26 V (53.6%), and the capacity retention rate remains 77.8% after 250 cycles. X-ray diffraction (XRD), transmission electron microscopy (TEM) and X-ray photoelectron spectroscopy (XPS) analysis showed that the introduction of sulfur induced the formation of a heterostructure of MoS_2_/MoS_3_ in the Mo_2_C lattice, which enhanced the conductivity and oxygen reduction/precipitation (ORR/OER) activity of the material. In addition, sulfur doping promotes the formation of highly conductive amorphous Li_2_O_2_ and effectively inhibits the accumulation of insulating ring Li_2_O_2_, thus significantly improving the cycle stability and energy efficiency of the battery. This study provides a new structural regulation strategy for the design of high efficiency lithium oxygen battery catalysts.

## Abstract 2

S@Mo_2_C cathode significantly enhances Li–O_2_ battery performance: specific capacity 3955 mA h g^−1^, overpotential 0.26 V, 77.8% capacity retention after 250 cycles. Sulfur doping induces MoS_2_/MoS_3_ heterostructure formation, enhances conductivity activity.

## Graphical abstract

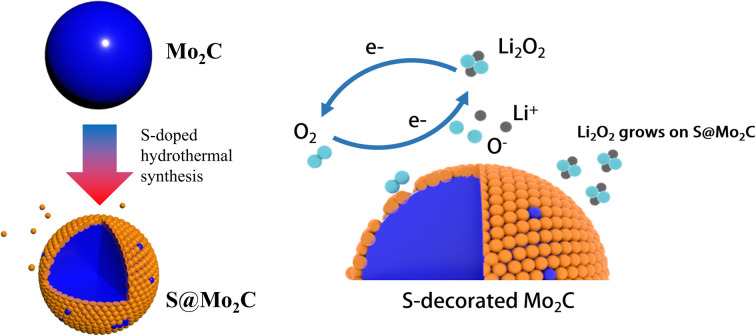

## 1 Introduction

The energy sector has undergone transformative changes aligned with modern developmental paradigms, driven by accelerated societal progress and escalating global environmental degradation. Among emerging energy storage technologies, lithium–oxygen (Li–O_2_) batteries have garnered substantial scientific interest due to their exceptional theoretical energy density of 11 586 mA h g^−1^ – approximately 3–5 times greater than conventional lithium-ion systems.^1,2^ This characteristic positions Li–O_2_ batteries as a highly promising next-generation energy storage solution.

However, the practical implementation of this technology faces critical challenges, primarily stemming from elevated overpotentials that impede reaction kinetics and compromise cycling efficiency.^3,4^ Current research identifies the insulating nature of lithium peroxide (Li_2_O_2_), the predominant discharge product, as a principal contributor to these high overpotentials. Although the more conductive lithium superoxide (LiO_2_) is also generated during the reaction process, its inherent instability prevents it from persisting as the primary discharge product.^5,6^ This limitation underscores the necessity for advanced catalytic materials capable of effectively decomposing Li_2_O_2_. Consequently, the development of high-performance catalysts has become a pivotal research focus, as such materials could substantially mitigate overpotential challenges and enhance battery performance through optimized reaction pathways.

Molybdenum carbide (Mo_2_C) is widely used as high effective catalysts. With abundant active sites, however, typical β-type Mo_2_C with a stable structure still does not show satisfactory performance in practice and needs more adjustment and optimization, and structural optimization is a very effective method.^7–10^ Metal sulfides are also widely used as electrode materials.^11,12^ The use of Mo_2_S as a catalyst in HER reactions has been widely investigated due to its excellent catalytic performance. Crystalline Mo_2_S and amorphous Mo_2_S_*x*_ are commonly used, however, the stacking layer structure causes it to exhibit a high impedance, resulting in a decrease of its conductivity, which implies a decrease in catalytic efficiency. The catalytic efficiency of Mo_2_S_*x*_ can be effectively improved by reconstructing the structure, which makes Mo_2_S_*x*_ feasible as a catalyst for Li–O_2_ batteries.^13,14^

While previous studies have established the catalytic potential of Mo_2_C- and MoS_2_-based cathodes in Li–O_2_ systems,^15–17^ critical limitations persist in addressing key electrochemical challenges. Conventional Mo_2_C cathodes often suffer from insufficient electrical conductivity and poor Li_2_O_2_ decomposition kinetics, typically manifesting in high charge overpotentials (>0.4 V) and rapid capacity fading (<50 cycles) due to parasitic side reactions.^8,18^ Although sulfur incorporation strategies (*e.g.*, MoS_2_ heterostructures) have been explored to enhance surface reactivity,^19^ most reported systems exhibit suboptimal phase stability under deep cycling conditions, with inevitable MoS_2_/Mo_2_C phase segregation leading to active site passivation.^20^ Furthermore, existing sulfur-doped architectures predominantly focus on single-phase modifications, neglecting the synergistic effects of multivalent Mo species (*e.g.*, Mo^3+^/Mo^4+^ redox couples) in regulating Li_2_O_2_ nucleation pathways.^21^

In this work, the S@Mo_2_C structure with efficient, conductive structure and active sites were optimized successfully by using commercial Mo_2_C with thioacetamide, and the S element was successfully doped during calcination, and used as the cathode to explore the synergistic effect of Li–O_2_ battery. As a result, the S-modified Mo_2_C structure can effectively improve the performance of the Li–O_2_ battery system with more excellent specific capacity (3955 mA h g^−1^), lower overpotential (0.26 V), and improved cycling performance (over 250 cycles) compared with the conventional commercial Mo_2_C.

In view of the reasons for the abnormally high specific capacity of our work: our analysis is composed of the following: Potential Factors Contributing to Enhanced Capacity in Lithium–Oxygen Battery Systems.

### 1.1 Material-specific reaction mechanisms

#### 1.1.1 Multielectron redox pathways

The S@Mo_2_C composite demonstrates catalytic capabilities in facilitating multielectron transfer processes during Li_2_O_2_ formation/decomposition cycles (eqn (1) and (2)). Sulfur doping enhances oxygen adsorption on the Mo_2_C substrate, thereby optimizing oxygen reduction reaction (ORR) and oxygen evolution reaction (OER) kinetics through improved intermediate stabilization.

#### 1.1.2 Heterophasic structural synergy

X-ray photoelectron spectroscopy (XPS) and X-ray diffraction (XRD) analyses confirm the formation of a MoS_2_/MoS_3_ hybrid phase post-sulfur incorporation (Fig. 4). This composite architecture exhibits enhanced electrical conductivity compared to pristine Mo_2_C, with electrochemical characterization revealing a reduced reaction overpotential (Δ*η* = 0.26 V). The synergistic phase interaction consequently improves the system's reversible capacity through optimized charge transfer efficiency.

### 1.2 Nanostructural optimization effects

#### 1.2.1 Two-dimensional charge transport enhancement

Transmission electron microscopy (TEM) characterization reveals the development of a 5 nm-thick nanomembrane surface layer on S@Mo_2_C (Fig. 3a). This ultrathin architecture facilitates rapid ion diffusion kinetics by reducing Li^+^ and O_2_ transport pathways while expanding the electrochemically active surface area.

#### 1.2.2 Crystalline defect engineering

Sulfur doping induces structural reorganization in the Mo_2_C lattice (Fig. 2), as verified by XPS analysis (Fig. 4e). The modified coordination environment generates abundant Mo^3+^ active sites, which promote homogeneous Li_2_O_2_ nucleation/decomposition processes through enhanced surface adsorption energetics.

## 2 Experimental section

### 2.1 Materials and chemicals

Commercial Mo_2_C (Sigma, 99.0%), deionized water, thioacetamide (Aladdin), lithium bis(trifluoromethanesulfony) imide (LiTFSI, Aladdin), tetraethylene glycol dimethyl ether (TEGDME, Aladdin), polyvinylidene fluoride (PVDF, Aladdin) and *N*-methylpyrrolidone (NMP, Aladdin) are all analytical pure.

### 2.2 Preparation of S-decorated Mo_2_C

Commercial Mo_2_C (120 mg) was added to 50 ml deionized water, and thioacetamide (636.76 mg) was further added. After electromagnetic stirring for 10 min, the mixture was placed in a 100 ml teflon-lined stainless steel autoclave, and heated to 473 K and the temperature maintained for 10 h. After cooling down, the mixture precursor was obtained. After centrifugation (7800 rpm) and dry (60 °C for 12 h), the black target specimen was obtained.

### 2.3 Preparation of Li–O_2_ cells

Commercial Mo_2_C (or S@Mo_2_C) was mixed with polyvinylidene fluoride (PVDF) and *N*-methylpyrrolidone (NMP) binder in a weight ratio of 8 : 1 : 1 to form a slurry, which was placed on carbon paper discs (14 mm diameter) and then dried in a vacuum oven at 120 °C for 12 h. The amount of mass loading is about 2 mg. The button cell used for electrochemical testing consisted of a cathode, 0.1 ml electrolyte (1 M LiTFSI in TEGDME) permeated to a glass fiber septum (Whatman GF/D microfiber filter paper, pore size 2.7 μm) and a lithium metal anode.

## 3 Results and discussion

**Fig. 1:**
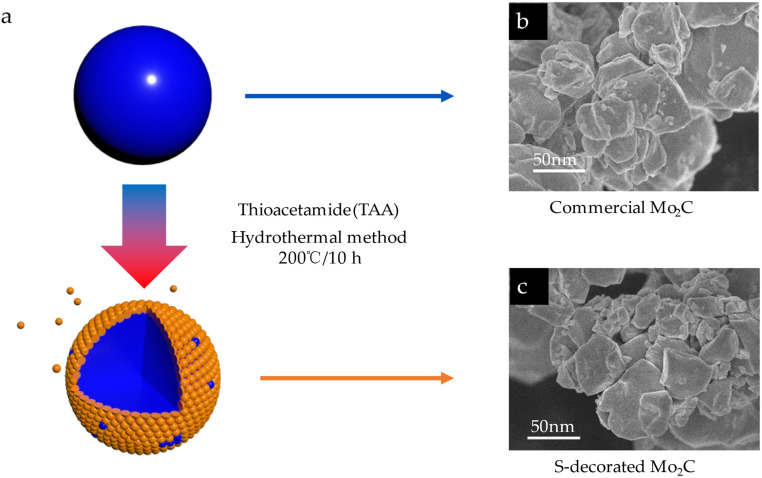
(a) Schematic synthesis process of S-decorated Mo_2_C; (b) the SEM image of commercial Mo_2_C; (c) the SEM image of S@Mo_2_C.

Fig. 1a shows the process of obtaining S-decorated Mo_2_C by doping with sulfur for commercial Mo_2_C by hydrothermal method. The commercial Mo_2_C (Fig. 1b) was added to deionized water and thioacetamide (TAA) was added to the mixture to obtain the precursor solution, which was further heated over 200 °C to obtain S-decorated Mo_2_C (Fig. 1c).

**Fig. 2:**
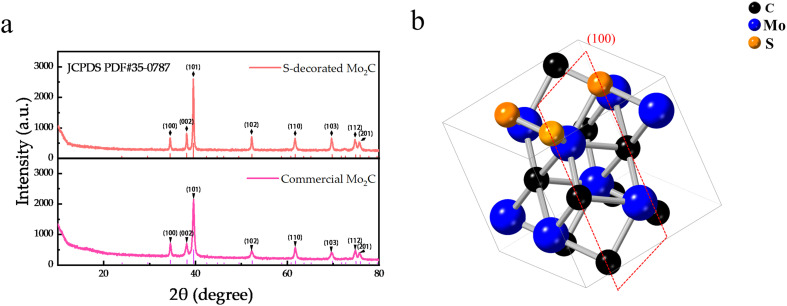
(a) XRD patterns of commercial Mo_2_C and S-decorated Mo_2_C; (b) crystal structure of S-decorated Mo_2_C.

Fig. 2a presents the X-ray diffraction (XRD) patterns of commercial Mo_2_C and S@Mo_2_C. Significant strong peaks are observed at 34.5, 38.0, 39.6, 52.3, 61.9, 69.8, and 75.0°, corresponding to the (100), (002), (101), (102), (110), (103), and (112) planes of the typical β-Mo_2_C crystal structure, respectively. Further analysis indicates that the original crystal structure is preserved after sulfur modification. Compared with the Mo_2_C sample before sulfidation, the diffraction peak intensities on the (002), (101), (102), (110), (103), and (112) planes have increased to varying degrees, and the peak widths have narrowed, suggesting an increase in grain size. This phenomenon can be attributed to the sulfur atom radius (0.104 nm) being larger than that of carbon (0.077 nm) during the sulfidation process, leading to lattice expansion and reconstruction of the crystal structure, thereby causing changes in the crystal diffraction characteristics. On the other hand, the decrease in the intensity of the (100) peak of Mo_2_C may be due to sulfur replacing carbon atoms on the (100) plane, forming MoS_2_ with a 2H structure, which reduces the number of Mo_2_C structures on the (100) plane (see Fig. 2b). The enhanced intensity of the (002) plane is attributed to sulfur doping optimizing the interlayer stacking of Mo_2_C, promoting the preferential growth of the S@Mo_2_C (002) plane.

**Fig. 3:**
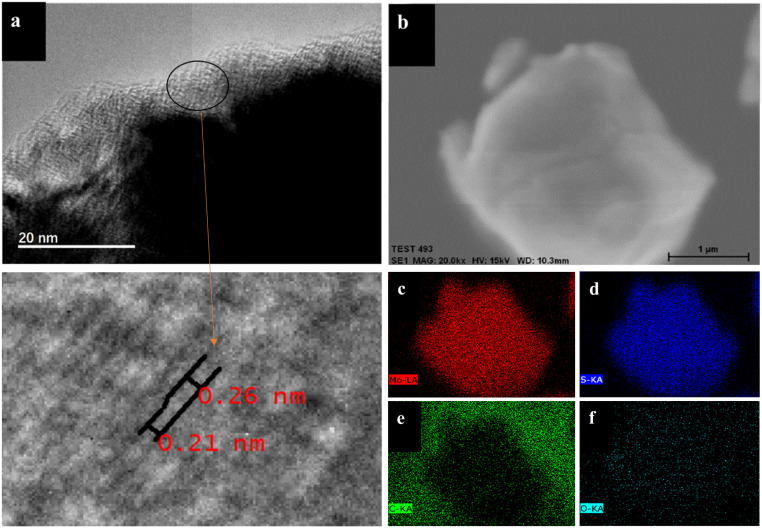
(a) The TEM and magnified STEM images of S-decorated Mo_2_C; (b–f) elemental mapping analysis of S-decorated Mo_2_C.

**Fig. 4:**
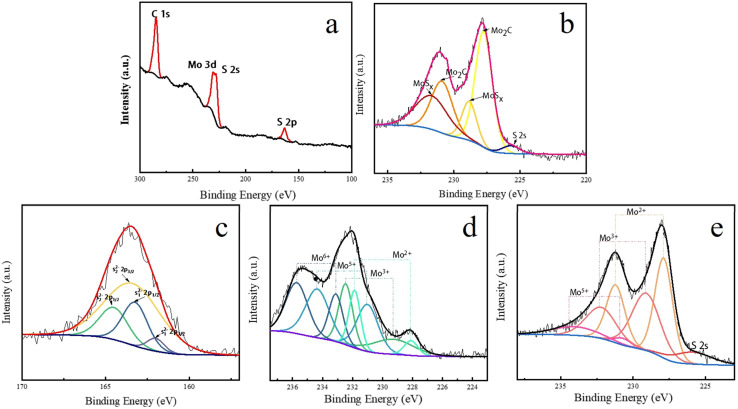
(a and b) XPS images of S@Mo_2_C sample; (c) XPS spectra of S element of S@Mo_2_C; (d and e) XPS images of different ion valences of Mo element in commercial Mo_2_C and S@Mo_2_C.

To deeply explore the microstructural characteristics of S-modified Mo_2_C, this study employed transmission electron microscopy (TEM) techniques for detailed observation and analysis. The high-resolution TEM image of S-modified Mo_2_C (Fig. 3a) reveals the uniform distribution of the S-modified Mo_2_C structure. The TEM image clearly shows a thin nanometer-thick film layer with a thickness of about 5 nanometers on the outer layer of Mo_2_C. By magnified scanning transmission electron microscopy (STEM) images, significant changes in the Mo_2_C lattice spacing can be observed, where the complete Mo_2_C lattice spacing distorts from 0.26 nanometers (corresponding to Mo_2_C (100), PDF#35-0787) to 0.21 nanometers (corresponding to MoS_2_ (006), PDF#73-1508). In the X-ray photoelectron spectroscopy (XPS) S 2p spectrum, the double peaks at 162.02 eV and 163.32 eV correspond to the 2p^3/2^ and 2p^1/2^ orbitals of s^2−^, confirming the presence of sulfur in the form of s^2−^ (Fig. 4c). Additionally, in the Mo 3d spectrum of XPS, the peaks at 228.85 eV and 231.8 eV shift, indicating the formation of Mo–S bonds. This result further verifies that the change in layer spacing is caused by the formation of MoS_2_ structures due to sulfur doping in the Mo_2_C lattice, consistent with the analysis results of the X-ray diffraction (XRD) spectrum. Fig. 3b shows the scanning electron microscopy-energy dispersive X-ray spectroscopy (SEM-EDX) surface scanning image of S-doped Mo_2_C, and Fig. 3c–f display the content and distribution of Mo, S, C, and O elements, respectively. Mo, S, C, and O elements are uniformly distributed throughout the sample, further confirming the formation of S@Mo_2_C. Notably, throughout the sample, especially in Fig. 3f, the distribution of O elements can be clearly observed, which may be due to the oxidation reaction of the sample with oxygen in the air under exposure conditions, leading to the formation of some MoO_*x*_. However, the MoO_*x*_ signal in the XPS analysis is weak, so the impact of MoO_*x*_ contamination on the experimental results can be ignored. Moreover, a trace amount of MoO_*x*_ may act as a protective layer to inhibit the decomposition of the electrolyte.

To further analyze the composition of the chemical states on the surface of S@Mo_2_C, X-ray photoelectron spectroscopy (XPS) was used. The XPS spectrum (Fig. 4) shows that S@Mo_2_C is comprised of Mo, C, O and S elements. From Fig. 4a, Mo 3d peaks and S 2s, S 2p peaks can be clearly observed, indicating that S element is present in large quantities of the S@Mo_2_C sample, with a mass fraction of 14.86% corresponding to Table 1, which is highly close to previous research.^22^ Strong peaks were observed at 227.75 eV (3d^5/2^) and 230.9 eV (3d^3/2^), and the binding energy characteristics were consistent with β-Mo_2_C. Compared with the typical Mo 3d peak (∼229 eV) of 2H–MoS_2_, the Mo 3d peak in S@Mo_2_C shifts 0.15 eV towards the high binding energy, which corresponds to the lattice expansion caused by the incorporation of S in the XRD pattern (Δ*d* = 1.36%). It is indicated that Mo and S may form a complex coordination structure of crystalline MoS_2_ and amorphous MoS_3_, MoS_*x*_.

**Table 1:** The atomic percent and mass percent of S@Mo_2_C

	C 1s	Mo 3d	S 2p
At%	79.89%	9.77%	10.34%
Wt%	43.05%	42.09%	14.86%

Fig. 4b shows the high-resolution spectrum of the binding energy in the range of 220–237.5 eV, where the presence of Mo_2_C and Mo_2_S_*x*_ with different valence Mo elements can be clearly observed. The peak of S 2 s is located at around 225.7 eV. Mo 3d exhibits strong peaks at 227.75 and 230.9 eV, respectively, showing a doublet with β-Mo_2_C characteristics.^23^ The Mo 3d peaks at 228.85 and 231.8 eV has shifted ∼0.15 eV compared with the normal value of 2H-MoS_2_ (229 eV), which can be certified by the characteristic double peaks of the Mo element of MoS_*x*_ in the Mo^4+^ state.^24^ As shown in the high-resolution S 2p spectrum (Fig. 4c), the peaks of S_1_^2−^ and S_2_^2−^ ligands at 162.02 and 163.32 eV corresponding to S_1_^2−^ 2p^3/2^ and S_1_^2−^ 2p^1/2^, respectively, can be observed. The ∼1.2 eV energy separation between S_1_^2−^ and S_2_^2−^ is the characteristic of S^2−^ in MoS_2_. Moreover, the peaks at 163.52 and 164.62 eV corresponding to S_2_^2^ 2p^3/2^ and S_2_^2−^ 2p^1/2^ are also observed, respectively. The energy separation of doublet is close to 1.2 eV, which is characteristic of S^2−^ in MoS_3_ species.^25^ Thus, the poorly crystalline “MoS_*x*_” in S@Mo_2_C is identified as a mixture of crystalline MoS_2_ and non-crystalline MoS_3_. The XPS spectra of Mo elements show the presence of different chemical valence states on the Mo surface, as shown in Fig. 4 (d, commercial Mo_2_C) and (e, S@Mo_2_C). The Mo–C bond in Mo_2_C contributes to the Mo^2+^ state and the partially low oxidation state of Mo^3+^, while other reports have shown that the Mo–O bond can also be assigned to Mo^3+^ and Mo^5+^ in MoO_2_, as well as Mo^6+^ in MoO_3_ due to air contamination.^26^ In Fig. 4d, Mo 3d^5/2^ peaked at 229.0 eV, and Mo 3d_3_ peaked at 232.1 eV, corresponding to Mo^3+^ (mainly MO–C bond). A weak peak at 235.5 eV was attributed to Mo^6+^ (MoO_3_) formed by surface oxidation. In Figure e, the Mo 3d^5/2^ peak migrates towards the low binding energy to 227.75 eV and the Mo 3 d^5/2^ peak is located at 230.9 eV, indicating a reduction in the oxidation state of Mo (Mo^3+^, Mo^2+^). The peak strength of Mo^6+^ decreased significantly (peak weakening at 235.5 eV), indicating that S doping inhibited surface oxidation and induced partial reduction of high-priced Mo (Mo^6+^, Mo^5+^) to low-priced Mo (Mo^3+^, Mo^2+^). The phenomena of lower valence states of eMo in Fig. 4c and d are both due to the incorporation of S, and the electronegativity of S is higher than that of C, resulting in S atoms attracting electrons from Mo and reducing the electron density of D orbital of Mo (increasing binding energy). Moreover, the introduction of S simultaneously provides electronic compensation by forming Mo–S bonds. It can be observed that the peaks of Mo^6+^ disappear, the intensity of Mo^5+^ peaks decreases, and the intensity of Mo^3+^ and Mo^2+^ peaks increases, indicating that Mo^6+^ and part of Mo^5+^ are reduced to lower Mo^3+^ and Mo^2+^ due to S doping. The appearance of a slight amount of S 2s peaks may be due to the oxidation of a slight amount of S^2−^ to SO_4_^2−^ during the preparation process.^27^

The plateau fraction of carbon (79.89%) was mainly derived from the carbon paper substrate used in electrode preparation (Fig. 3c SEM-EDX surface scan shows uniform distribution of carbon). Sulfur doping (10.34 at%) leads to a decrease in molybdenum content (9.77 at%) due to the partial substitution of sulfur atoms for carbon atoms in the Mo_2_C lattice to form the Mo_2_C_1−*x*_ S_*x*_ hybrid structure. The reduction of Mo–C bond strength in the XPS analysis (Fig. 4) further supports this substitution mechanism.

**Fig. 5:**
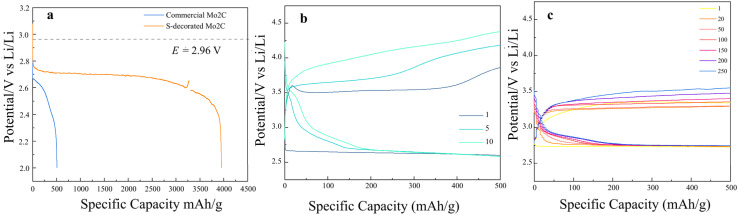
(a) Voltage profile of the full discharge for commercial Mo_2_C and S@Mo_2_C in Li–O_2_ batteries; (b) charge and discharge curves of commercial Mo_2_C at a current density of 100 mA g^−1^; (c) charge and discharge curves of S@Mo_2_C at a current density of 100 mA g^−1^.

To verify the properties of kinetics reaction of S@Mo_2_C, a coin-type Li–O_2_ battery consisting of a lithium metal anode was used for the electrochemical test. The galvanostatic full discharge curves of Mo_2_C and S@Mo_2_C at a 100 mA g^−1^ current density with 0.1 mV s^−1^ scan rate from 2.0 to 4.5 V (*vs.* Li/Li^+^) were obtained in Fig. 5a. Obviously, the coexistence of S and Mo_2_C as a cathode for Li–O_2_ battery presents the largest capacity (3955 mA h g^−1^), which shows a much higher specific capacity compared to commercial Mo_2_C cathode (508 mA h g^−1^). Moreover, the discharge overpotential is significantly decreases from 560 mV to 260 mV for the S@Mo_2_C cathode, indicating that the more completely ORR (oxidation–reduction reaction) have occurred at the positive electrode and the discharge products have grown more fully on the S@Mo_2_C cathode than commercial Mo_2_C. S@Mo_2_C with better electrical conductivity and ORR performance can be attributed to lower overpotential and more fully grown discharge products.^28^

The discharge and charge curves of Mo_2_C and S@Mo_2_C cathodes with a cut off capacity of 500 mA h g^−1^ at a current density of 100 mA g^−1^ were investigated in Fig. 5b and c. It can be observed clearly that commercial Mo_2_C cathode presents a good OER (oxygen evolution reaction) kinetics in Fig. 5b. As the charge/discharge proceeds, the overpotential continues to increase after 5 and 10 cycles rapidly (∼1.5 V overpotential, ∼630 mV increased after 10 cycles), while the round-trip efficiency degrades from 75.1% down to 63.2%. Compared with commercial Mo_2_C, S@Mo_2_C cathode presents a better, stable and reversible charge/discharge characteristic in Fig. 5c. The charge/discharge curves from the 1st to even 250th cycle of S@Mo_2_C presents a great overpotential difference (∼0.8 V overpotential, ∼250 mV increased after 250 cycles) and a smooth charge/discharge plateau with little overpotential difference (3.7% degradation of round-trip efficiency, 77.8% remained after 250 cycles). Lower overpotential allows the discharge products Li_2_O_2_ decomposing effectively, OER occurring more fully at the cathode and avoiding the generation of by-products effectively.^29,30^ Besides, charging plateaus below 3.5 V is very beneficial to the cycling performance for the Li–O_2_ battery, as carbon materials decompose above 3.8 V.^31^ In order to better reveal the mechanism, the charge/discharge characteristics of S@Mo_2_C cathode will be analyzed next. A direct comparison with the previous material properties is tabulated as shown in Table 2 below.

**Table 2:** Material performance comparison

Catalyst	Specific capacity (mA h g^−1^)	Overpotential (V)	Cycle stability
**S@Mo**_**2**_ **C**	**3955**	**0.26**	**250 cycles**
Mo_2_C	508	0.56	<50 cycles
Co_3_O_4_HPNT	4146	0.099	40
Mo_2_C/MoO_2_@RGO	2365	0.56	
Mo_2_C/C	7500	1.2	100 cycles
MoN	7400	0.19	

**Fig. 6:**
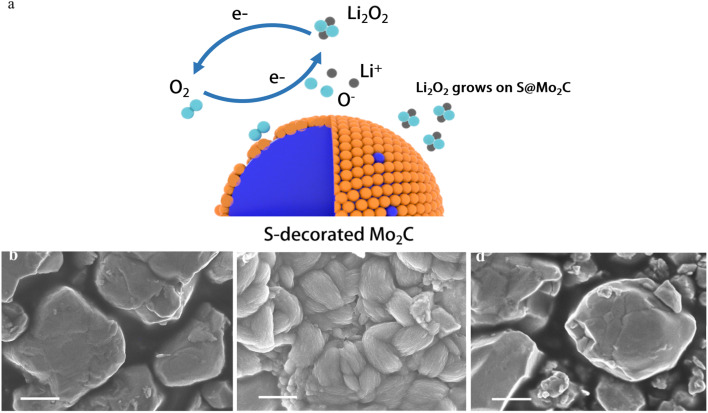
(a) The schematic diagram of S@Mo_2_C electrode Li–O_2_ battery; (b) pristine S@Mo_2_C cathode with uniform nanoparticles. (c) post-discharge: toroidal Li_2_O_2_ and amorphous Li_2_O_2_ deposited on the surface. (d) Post-recharge: complete decomposition of Li_2_O_2_, restoring the cathode morphology. The (c) and (d) two figures are TEM images after 250 cycles.

In lithium–oxygen batteries, S@Mo_2_C is used as the cathode material, and its charge–discharge process is monitored using a scanning electron microscope (SEM), corresponding to the different states shown in Fig. 6a (Fig. 6b–d). The charge–discharge reactions of the battery follow the following chemical eqn (1) and (2):^32^

1 Discharge: 2Li^+^ + O_2_ + 2e^−^ → Li_2_O_2_

2 Charge: Li_2_O_2_ → 2Li^+^ + O_2_ + 2e^−^

The initial state of S@Mo_2_C presents a flat and undamaged surface, as shown in Fig. 6b. As the discharge process progresses, a small-sized, high-crystallinity annular film forms on the cathode surface, a phenomenon clearly visible in Fig. 6c. This annular film is considered the typical discharge product Li_2_O_2_. Under normal circumstances, the annular Li_2_O_2_ is the main factor causing the high overpotential of lithium–oxygen batteries.^33^ However, during the recharging process, the decomposition of Li_2_O_2_ is observed, and S@Mo_2_C returns to its original flat and undamaged state, which is very similar to Fig. 6b, indicating that S@Mo_2_C can return to its initial form after charging. After 250 cycles of electrochemical stability testing, the capacity retention rate reaches 77.8%, and the overpotential only increases by 250 mV. The low overpotential (0.8 V) indicates a high reversibility of Li_2_O_2_ decomposition, reducing structural stress, thus indicating that the nanosheets did not undergo structural collapse or peeling during the charge–discharge cycles, showing good crystal structure stability. This suggests that not only is annular Li_2_O_2_ formed, but also amorphous Li_2_O_2_ with higher conductivity. Under high current density conditions, the formation of small annular Li_2_O_2_ is more easily observed.^34^ Typically, amorphous Li_2_O_2_ promotes rapid electron tunneling during the charging process, resulting in rapid and efficient decomposition of Li_2_O_2_.^35,36^ Interestingly, although S doping may adversely affect the number of active sites on the Mo_2_C cathode, leading to an increase in cathode impedance, S doping also improves the quality of the effective active sites, significantly enhancing the conductivity of S@Mo_2_C.^37^ High-quality active sites may also more effectively break the O–O bond, thereby significantly reducing the activation energy required for the reaction. While experimental evidence including XPS analysis of Mo^4+^/Mo^6+^ ratios strongly supports the proposed mechanism of Mo valence tuning and O–O bond activation, future density-functional theory (DFT) calculations will contribute to a comprehensive understanding of the mechanism. Such computational studies can quantitatively verify synergistic effects between Co/Mo sites, accurately map reaction pathways (*e.g.*, O–O cleavage energies), and reveal electronic structure modifications at the atomic level. Nevertheless, the consistency between our structural characterization (XRD, XPS, TEM) and the excellent electrochemical properties (low overpotential, high stability) provides a reliable empirical validation of the material's design strategy.^38–40^

## 4 Conclusions

In this study, we successfully modified commercial Mo_2_C (S@Mo_2_C) cathode material by sulfur doping strategy, and prepared sulfur modified Mo_2_C (S@Mo_2_C) cathode material. The system characterization indicated that the introduction of sulfur not only optimized the crystal structure of Mo_2_C and formed a heterogeneous interface of MoS_2_/MoS_3_, but also significantly increased the conductivity and catalytic activity of the material. Electrochemical tests show that the S@Mo_2_C-based lithium–oxygen battery achieves a high specific capacity of 3955 mA h g^−1^ at a current density of 100 mA g^−1^, reduces the charge and discharge overpotential to 0.26 V, and exhibits excellent cycle stability (capacity retention rate of 77.8% after 250 cycles). The mechanism study shows that sulfur doping can improve battery performance in the following ways: (1) enhance electron transport capacity and promote ORR/OER reaction kinetics; (2) induce the formation of highly conductive amorphous Li_2_O_2_ and inhibit the irreversible accumulation of insulating ring Li_2_O_2_; (3) optimize the distribution of active sites and reduce the reaction energy barrier. In addition, the S@Mo_2_C stable charge and discharge platform (<3.5 V) effectively avoids the decomposition of carbon-based materials, further extending the battery life. This study provides an important theoretical and experimental basis for the development of efficient and long-lived lithium–oxygen battery catalysts. Subsequent studies can be extended to explore the generalizability of the sulfur doping strategy in other transition metal carbide systems and large-scale preparation techniques, while incorporating density-functional theory (DFT) modeling in order to establish a clear conformational relationship and guide the further optimization of the bimetallic catalysts.

## Author contributions

Yanhong Ding was responsible for the experimental design and writing the first draft of the paper, Zhichao Gao was responsible for writing or revising the chapters, Rongpeng Lin was responsible for the experimental part, Yong Cao assisted in completing the experiments, Haoyang Liu assisted in completing the experiments or data analysis, Yulin Zhou assisted in completing the data analysis, Haifeng Xu was responsible for finding the related papers on Li–O_2_, Jiayi Liu was involved in the XRD part of the revisions, Fangqi Ren was involved in the XPS part of the revisions, Yirong Zhu was responsible for the paper submission, response to review comments and communication with journals.

## Conflicts of interest

On behalf of all authors, the corresponding author states that there is no conflict of interest.

## Data Availability

The datasets generated during and/or analysed during the current study are available from the corresponding author on reasonable request.
